# Receptor conversion in distant breast cancer metastases

**DOI:** 10.1186/bcr2645

**Published:** 2010-09-23

**Authors:** Laurien DC Hoefnagel, Marc J van de Vijver, Henk-Jan van Slooten, Pieter Wesseling, Jelle Wesseling, Pieter J Westenend, Joost Bart, Cornelis A Seldenrijk, Iris D Nagtegaal, Joost Oudejans, Paul van der Valk, Petra van der Groep, Elisabeth GE de Vries, Elsken van der Wall, Paul J van Diest

**Affiliations:** 1 Department of Pathology, University Medical Center Utrecht, Heidelberglaan 100, 3584 CX Utrecht, The Netherlands; 2 Department of Pathology, Academic Medical Center, Meibergdreef 9, 1105 AZ Amsterdam, The Netherlands; 3 Department of Pathology, Medical Center Alkmaar, Wilhelminalaan 12, 1815 JD Alkmaar, The Netherlands; 4 Department of Pathology, Medical Center Zaandam, Koningin Julianaplein 58, 1502 DV Zaandam, The Netherlands; 5 Department of Pathology, Radboud University Medical Centre, Geert Grooteplein Zuid 10, 6525 GA Nijmegen, The Netherlands; 6 Department of Pathology, Canisius Wilhelmina Hospital, Weg door Jonkerbos 100, 6532 SZ Nijmegen, The Netherlands; 7 Department of Pathology, the Netherlands Cancer Institute, Plesmanlaan 121, 1066 CX Amsterdam, The Netherlands; 8 Laboratory for Pathology, Laan van Londen 1800, 3317 DA Dordrecht, The Netherlands; 9 Department of Pathology, University Medical Center Groningen, Hanzeplein 1, 9700 RB Groningen, The Netherlands; 10 Department of Pathology, St. Antonius Hospital, Koekoekslaan 1, 3435 CM Nieuwegein, The Netherlands; 11 Department of Pathology, Diakonessenhuis, Bosboomstraat 1, 3582 KE Utrecht, The Netherlands; 12 Department of Pathology, Free University Medical Center, De Boelelaan 1117, 1081 HV Amsterdam, The Netherlands; 13 Division of Internal Medicine and Dermatology, University Medical Center Utrecht, Heidelberglaan 100, 3584 CX Utrecht, The Netherlands; 14 Department of Medical Oncology, University Medical Center Groningen, Hanzeplein 1, 9700 RB Groningen, The Netherlands

## Abstract

**Introduction:**

When breast cancer patients develop distant metastases, the choice of systemic treatment is usually based on tissue characteristics of the primary tumor as determined by immunohistochemistry (IHC) and/or molecular analysis. Several previous studies have shown that the immunophenotype of distant breast cancer metastases may be different from that of the primary tumor (receptor conversion), leading to inappropriate choice of systemic treatment. The studies published so far are however small and/or methodologically suboptimal. Therefore, definite conclusions that may change clinical practice could not yet be drawn. We therefore aimed to study receptor conversion for estrogen receptor alpha (ERα), progesterone receptor (PR), and human epidermal growth factor receptor 2 (HER2) in a large group of distant (non-bone) breast cancer metastases by re-staining all primary tumors and metastases with current optimal immunohistochemical and in situ hybridization methods on full sections.

**Methods:**

A total of 233 distant breast cancer metastases from different sites (76 skin, 63 liver, 43 lung, 44 brain and 7 gastro-intestinal) were IHC stained for ERα, PR and HER2, and expression was compared to that of the primary tumor. HER2 *in situ *hybridization (ISH) was done in cases of IHC conversion or when primary tumors or metastases showed an IHC 2+ result.

**Results:**

Using a 10% threshold, receptor conversion by IHC for ERα, PR occurred in 10.3%, 30.0% of patients, respectively. In 10.7% of patients, conversion from ER+ or PR+ to ER-/PR- and in 3.4% from ER-/PR- to ER+ or PR+ was found. Using a 1% threshold, ERα and PR conversion rates were 15.1% and 32.6%. In 12.4% of patients conversion from ER+ or PR+ to ER-/PR-, and 8.2% from ER-/PR- to ER+ or PR+ occurred. HER2 conversion occurred in 5.2%. Of the 12 cases that showed HER2 conversion by IHC, 5 showed also conversion by ISH. One further case showed conversion by ISH, but not by IHC. Conversion was mainly from positive in the primary tumor to negative in the metastases for ERα and PR, while HER2 conversion occurred equally both ways. PR conversion occurred significantly more often in liver, brain and gastro-intestinal metastases.

**Conclusions:**

Receptor conversion by immunohistochemistry in (non-bone) distant breast cancer metastases does occur, is relatively uncommon for ERα and HER2, and is more frequent for PR, especially in brain, liver and gastro-intestinal metastases.

## Introduction

With 1,000,000 new cases causing 375,000 deaths worldwide per year, breast cancer is the leading cause of female cancer death worldwide [1]. Early detection, optimal surgery and adjuvant therapy are the key strategies to improving prognosis. Nevertheless, about one third of patients will develop distant metastases and eventually die of the disease. Patients who develop distant metastases usually undergo systemic therapy with chemotherapy, hormonal therapy and/or human epidermal growth factor receptor 2 (HER2) targeted therapy. Choice of therapy is currently personalized on the basis of the immunophenotype of the primary tumor, since distant metastases are often not biopsied, partly because of limited accessibility of these metastases, but also because it is not considered necessary for further therapeutic decision making.

However, previous studies [2-23] have indicated that receptor status of breast cancer metastases may differ from the primary tumor, generally denoted "receptor conversion". The published studies suggest that, compared to the primary tumors, estrogen receptor (ERα) and progesterone receptor (PR) are more frequently negative in distant metastases, whereas HER2 is more often positive. These observations, if confirmed, have important clinical consequences, since this would mean that a number of patients are withheld adequate systemic treatment for their metastases. In addition, if immunophenotype conversion would occur in high frequency, this would make it clinically very relevant to biopsy (even difficult to access) distant metastases to assess hormone receptor and HER2 status. An alternative for taking biopsies could be molecular imaging methods like positron emission tomography (PET) and single photon emission computed tomography (SPECT) that are currently being developed to functionally assess immunophenotype of breast cancer metastases [24-26].

Unfortunately, the previous conversion studies suffer from several limitations: small size (six studies contain <30 cases and eight studies ≤62 cases) [2,3,6,7,9,10,12-15,18-20,22], only one metastatic site studied [9,18], using a ligand-binding assay [2,3,7,10], which, especially in the case of metastases, may be biased by low cellularity and contamination by nonmalignant cells, inclusion of decalcified bone metastases [2,4,6,8-10,12,16,19,20,22] that may give rise to false negative immunohistochemistry, extraction of original immunohistochemistry results from the pathology report instead of repeating the staining [3,5,8,10,11,23], and/or use of tissue arrays [21] which may introduce sampling bias. Therefore, the available data may not be sufficiently reliable to change current clinical practice, although several guidelines already advise to rebiopsy distant metastases when possible [27,28]. Consequently, for most patients with metastatic breast cancer hormone receptor and HER2 status in the primary tumor are still used to guide therapy.

We have now performed a large study analyzing metastases from different sites, while restaining metastases and primary tumors side-by-side with optimal current immunohistochemical methods for ERα, PR and HER2 on full sections to asses the conversion rate of ERα, PR and HER2 status in distant metastases compared to the primary breast carcinomas.

## Materials and methods

### Patients

Two hundred and thirty-three primary breast carcinomas and corresponding metachronous non-bone distant metastases from female patients were obtained from the departments of pathology of the University Medical Center Utrecht, the Academic Medical Center Amsterdam, the Radboud University Nijmegen Medical Centre, the Canisius Wilhelmina Hospital Nijmegen, the Netherlands Cancer Institute Amsterdam, the Medical Center Alkmaar, the Medical Center Zaandam, the University Medical Center Groningen, the St. Antonius Hospital Nieuwegein, the Diakonessenhuis Utrecht, the Free University Medical Center Amsterdam, and the Laboratory for Pathology Dordrecht, all in The Netherlands. Original diagnoses were made between January 1985 and March 2009, and these cases comprised all the paired cases that could be retrieved from the participating labs during this period, minimizing selection bias. All histological specimens had been fixed for 12 to 24 hours in neutral buffered formaldehyde. The vast majority of primary specimens were paraffin blocks of breast or lumpectomies, except for 17 cases core biopsies from the primary tumors were used (no cytology). For 11 cases this information was not available. The sites of the distant metastases are shown in Table 1. Use of anonymous or coded left over material for scientific purposes is part of the standard treatment contract with patients in hospitals in The Netherlands [29]. Ethical approval was not required.

**Table 1:** Clinicopathologic characteristics of 233 invasive breast cancer patients studied for receptor conversion in distant metastases

Feature	Grouping	N or value	%
Age (years)	Mean	53.9	
	Range	25 to 93	

Tumor size (cm)	≤2	73	31.3
	> 2 and ≤5	80	34.3
	> 5	12	5.2
	Not available	68	29.2

Histologic type	Invasive ductal cancer	192	82.4
	Invasive lobular cancer	20	8.6
	Others	20	8.6
	Not available	1	0.4

Histologic grade	1	8	3.4
	2	61	26.2
	3	161	69.1
	Not available	3	1.3

MAI (per 2 mm^2^)	Mean	25	
	Range	0 to 172	
	≤12	71	30.5
	≥13	156	67.0
	Not available	6	2.5

Lymph node status	Positive	119	51.1
	Negative	81	34.8
	Not available	33	14.2

Site of distant metastases	Brain	44	18.9
	Lung	43	18.5
	Liver	63	27.0
	Skin	76	32.6
	Gastro-intestinal	7	3.0

For each case, hematoxylin-eosin stained slides of the paraffin blocks were reviewed by a single pathologist (PJvD) to confirm the presence of malignancy in tumor samples.

Histologic type was assessed according to the World Health Organization. Histologic grade was assessed according to the Nottingham modification of the Bloom-Richardson system, applying standardized mitotic counts [30]. Clinicopathologic characteristics are shown in Table 1.

### Immunohistochemistry

Immunohistochemical analysis was carried out on 4-μm sections. We did not use the tissue microarray approach, to avoid sampling bias due to tumor heterogeneity. All primary tumors and metastases were restained by the same person (LCDH) according to the same protocol to allow optimal pair-wise comparisons. For all stainings, slides were deparaffinized in xylene and rehydrated in decreasing ethanol dilutions. Endogenous peroxidase activity was blocked with H_2_O_2 _in phosphate buffered saline (PBS) followed by antigen retrieval. For ERα and HER2, antigen retrieval was performed in an autoclave with the slides placed in an EDTA buffer, pH = 9. For PR antigen retrieval was performed in citrate buffer, pH = 6 (20 minutes, 100°C). A cooling off period of 30 minutes preceded the incubation (60 minutes, room temperature) with the primary antibodies.

Mouse monoclonal antibodies used were: ERα (M7047, 1:80, DAKO, Glostrup, Denmark), PR (M3569, 1:50, DAKO) and HER2 (RM-9103-S, 1:100, Neomarkers, Lab Vision Corporation, Fremont, California, USA). For detection of the primary antibodies a poly HRP anti Mouse/Rabbit/Rat IgG (ready to use; Powervision, Immunovision Technologies, Brisbane, California, USA) was used. Between steps, slides were washed with PBS. Finally, peroxidase activity was developed with diaminobenzidin, slides were lightly counter-stained with hematoxylin, dehydrated in increasing alcohol dilutions and cover slipped. Appropriate negative and positive controls were used throughout. We regularly participate in EQA schemes to monitor our performance with these routine antibodies.

If HER2 status differed between primary tumor and metastases, or when either primary tumor or metastasis were IHC 2+ (see below), silver *in situ *hybridization (SISH) analysis [31] was performed with a fully automated technique (INFORM, Ventana Medical Systems, Tucson, AZ, USA) according to the manufacturer's guidelines.

Scoring of IHC slides was performed by one observer (PJvD) in random order, blinded to other data in the paired samples. For ERα and PR, the percentage of positively stained nuclei was estimated. In primary tumor samples, the adequacy of staining was checked by also evaluating the normal breast parenchyma when present. Samples with 10% or more immunopositive malignant cells were classified as ERα- or PR positive as usual [8,11,12]. In order to also comply with the most recent ASCO guidelines [32], we also used the 1% threshold that is now widely used in the USA. HER2 expression was scored using the DAKO scoring system as 0, 1+, 2+ and 3+ according to standardized criteria [33], considering 3+ cases as positive. SISH results were evaluated by one observer (MJvdV) according to the manufacturer's instructions blinded to other data in the paired samples and immunohistochemistry results. According to the ASCO/CAP guidelines [34], tumors with <6 HER2 copies/tumor cell nucleus were scored as HER2 non-amplified; and tumors with 6 or more HER2 copies/tumor cell nucleus were scored as HER2 amplified.

### Statistical analysis

Percentages of nuclei expressing ERα and PR in primary tumors and their metastases were compared by paired *t*-test (SPSS). The frequency of receptor expression (positive vs negative) in the primary tumors and distant metastases was calculated. Percentages of conversion were calculated for the whole group, and for subgroups of metastatic sites (10% threshold for ERα and PR only). As steroid receptor conversion is especially important if a patient converts from ER+ or PR+ to ER-/PR-, or from ER-/PR- to ER+ or PR+, we calculated the percentages for these conversions as well. Conversion rates for the different distant sites (10% threshold for ERα and PR) were compared by chi-square test.

## Results

**Figure 1:**
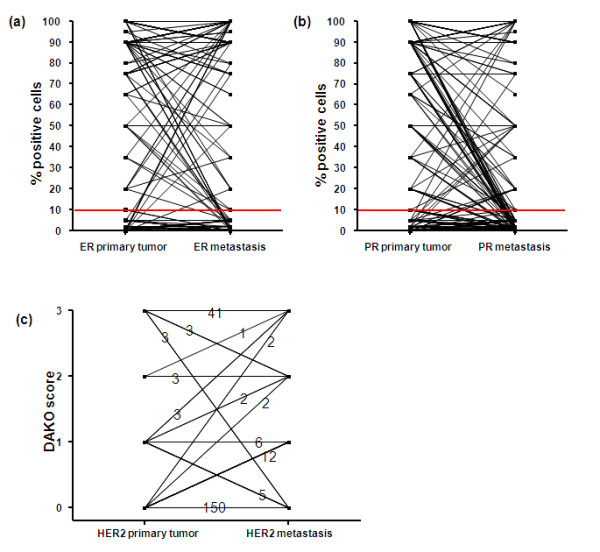
**Immunophenotype for ERα (a), PR (b) and HER2 (c) in 233 primary breast tumors and their corresponding metastases**.

The percentage of nuclei expressing ERα or PR was generally lower in the distant metastases than in the primary tumor (Figure 1), but significance was only reached for PR (*P *<0.001). Receptor conversion exceeding the threshold of 10% occurred for ERα in 10.3% and for PR in 30.0% of the patients (Table 2). Such conversion was mainly from positive to negative: 10.7% of the patients converted from ER+ or PR+ to ER-/PR-, and 3.4% from ER-/PR- to ER+ or PR+. Receptor conversion exceeding the threshold of 1% occurred for ERα in 15.1% and for PR in 32.6% of the patients (Table 2), while 12.4% of the patients converted from ER+ or PR+ to ER-/PR-, and 8.2% from ER-/PR- to ER+ or PR+.

**Table 2:** HER2 and ERα, PR expression by immunohistochemistry in paired primary breast cancers and their distant metastases

		Distant metastases					
**Primary tumor**	**Status**	**-**		**+**		Total	
HER2	-	180 (77.2%)		6 (2.6%)		186	
	+	6 (2.6%)		41 (17.6%)		47	

		10% threshold			1% threshold		
			
		-	+	Total	-	+	total

ERα	-	79 (33.9%)	7 (3.0%)	86	47 (20.2%)	12 (5.2%)	59
	+	17 (7.3%)	130 (55.8%)	147	23 (9.9%)	151 (64.8%)	147
PR	-	92 (39.5%)	12 (5.1%)	104	33 (14.2%)	27 (11.6%)	60
	+	58 (24.9%)	71 (30.5%)	129	49 (21.0%)	124 (53.2%)	173

For ERα and PR, data are shown using both the traditional 10% and new ASCO 1% thresholds.

HER2, human epidermal growth factor receptor 2; ERα, estrogen receptor alpha; PR, progesterone receptor.

Receptor conversion for ERα and PR (10% threshold) seemed to occur especially in liver (ERα 12.7%, PR 41.2%) and brain metastases (ERα 13.7%, PR 36.3%) (Table 3). For PR, conversion was significantly more often seen for brain, liver and gastro-intestinal metastases (*P *= 0.04).

**Table 3:** Receptor conversion for ERα, PR (10% threshold) and HER2 in distant breast cancer metastases according to site

		% conversion		
		
		ERα	PR	HER2
	N	N (%)	N (%)	N(%)
**Brain**	44	6 (13.7)	16 (36.3)*	1 (2.3)
**Lung**	43	4 (9.4)	8 (18.6)	2 (4.7)
**Liver**	63	8 (12.7)	26 (41.2)*	6 (9.5)
**Skin**	76	5 (6.6)	17 (22.3)	2 (2.6)
**Gastro-intestinal**	7	1 (14.3)	3 (42.9)*	1 (14.3)

* statistically significantly more often than for lung and skin metastases (*P *= 0.04, chi-square test).

ERα, estrogen receptor alpha; PR, progesterone receptor; HER2, human epidermal growth factor receptor 2.

For HER2, receptor conversion by IHC occurred in 5.2% of patients, about half of them from negative to positive and the other half from positive to negative (Table 2). Receptor conversion for HER2 seemed to occur especially, although not significantly, in liver metastases (9.5%) (Table 3). Of the 12 cases that showed HER2 conversion by IHC, 5 showed also conversion by SISH. One further case showed conversion by ISH, but not by IHC (Table 4).

**Table 4:** Silver *in situ *hybridization results for breast cancer cases showing HER2 receptor conversion in distant metastases by immunohistochemistry or 2+ scores by immunohistochemistry in either the primary tumor or the metastasis

Case	HER2 (IHC) primary tumor	HER2 (SISH) primary tumor	HER2 (IHC) metastasis	HER2 (SISH) metastasis	Metastatic site
1	0	No amplification	2+	No signal	lung
2	0	No amplification	2+	No signal	liver
3	0	High amplification	3+	High amplification	liver
4	0	Low amplification	3+	Low amplification	skin
5	1+	No amplification	2+	High amplification	lung
6	1+	No amplification	2+	No amplification	skin
7	1+	No amplification	3+	High amplification	skin
8	1+	No amplification	3+	High amplification	liver
9	2+	High amplification	2+	High amplification	liver
10	2+	High amplification	2+	High amplification	liver
11	2+	Low amplification	2+	Low amplification	liver
12	2+	Low amplification	3+	High amplification	liver
13	2+	Low amplification	3+	High amplification	liver
14	3+	High amplification	0	No amplification	liver
15	3+	High amplification	0	No amplification	liver
16	3+	High amplification	0	No amplification	gastro-intestinal
17	3+	High amplification	2+	High amplification	brain
18	3+	High amplification	2+	No signal	lung
19	3+	High amplification	2+	High amplification	lung

HER2, human epidermal growth factor receptor 2; IHC, immunohistochemistry; SISH, silver in situ hybridization.

**Figure 2:**
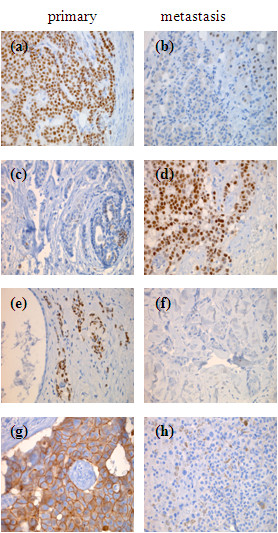
**Examples of receptor conversion in distant metastases of primary breast cancers in the same patient**. ERα positive primary tumor **(a) **with ERα negative liver metastasis that bears ERα expression in hepatocytes **(b)**. ERα negative primary tumor **(c) **with ERα positive brain metastases **(d)**. PR positive primary tumor **(e) **with PR negative skin metastasis **(f)**. HER2 positive (3+) primary tumor **(g) **with HER2 negative liver metastasis **(h)**.

Examples of conversion of ERα, PR and HER2 from primary breast cancers to distant metastases are shown in Figure 2.

## Discussion

Previous studies have shown that distant breast cancer metastases may show receptor conversion, potentially leading to inappropriate choices of systemic treatment in these patients. These previous studies however suffered from several limitations not allowing the researchers to draw definite conclusions that may change clinical practice. We therefore set out to reevaluate receptor conversion in a large group of non-bone distant breast cancer metastases using optimal methodology. Receptor conversion by IHC for ERα, PR and HER2 occurred in 10.3%, 30.0% and 5.2% of patients, respectively, using the traditional 10% threshold. When using the new 1% ERα and PR threshold according to the ASCO guidelines, conversion rates were even higher at 15.1% and 32.6%.

Previous studies (all using the 10% threshold when indicated) reported ERα receptor conversion rates from 12% to 54%, clearly higher than in the present study. The explanation for this finding may be that previous ERα studies did not restain both the primary and metastatic lesions [3,5,8,10,11], used ligand-binding assays [2,3,7,10] (where the result is influenced by differences in the percentage of non-tumor cells in the samples), or included bone metastases that may suffer from false negative IHC results due to decalcification [2,4,6,8-10,12]. In addition, with 233 cases our series is much larger than most previous ERα studies. Only two previous studies report on comparable numbers of cases (200 and 211, respectively), but in these studies the original immunohistochemistry results from the pathology report were used instead of renewed stainings. Previous studies (all using the 10% threshold when indicated) reported PR conversion rates from 28% to 61%, again higher than in the present study. The explanation for this may be similar to what has been mentioned above: previous studies did not restain both the primary and metastatic lesions [5,8,10,11], used ligand-binding assays [2,7,10], or included bone metastases [2,6,8-10,12]. Further, our series is much larger with 233 compared to most previous PR studies with 9 to 59 cases. There were two previous studies with respectively 173 and 211 cases, but these used the original immunohistochemistry results from the pathology report instead of renewed stainings.

Conversion for ERα and PR was mainly from positive in the primary tumor to negative in the metastases as has been described before [2,4,6,8,10-12]. This finding may well be explained by clonal selection of less differentiated receptor negative cells during the metastatic process, for example, elicited by adjuvant hormonal treatment [12,35,36]. However, in a few cases conversion from negative in the primary tumor to positive in the metastases occurred. This phenomenon has also been described before [2,3,8,10,11], but is more difficult to explain. Although false negative primary tumor results cannot be fully excluded, we also assessed the adequacy of staining by analysis of staining of epithelial cells in the normal ducts and lobules as an internal control. Perhaps in these cases small receptor positive clones within the primary tumor preferentially metastasized [37]. Alternatively, this phenomenon could be a result of genetic drift during tumor progression [38]. Previous studies reported HER2 immunophenotype conversion rates from 0% to 58.3%, which is generally higher than the 5.2% conversion rate we have found in the present study. As mentioned above, previous HER2 studies did not restain both the primary and metastatic lesions [10,11,23], used TMAs [21] or included bone metastases [10,12,16,19,20,22]. Further, our series is much larger with 233 cases compared to most previous studies with 12 to 211 cases, and in the only larger study (*N *= 382) restaining was not performed. By SISH, only half of these IHC conversions were accompanied by a difference in HER2 gene amplification status. Therefore, for only 6 out of the 233 patients (3%) a "true" conversion on the gene level of HER2 status between primary tumor and distant metastasis could be demonstrated. The fact that scoring of HER2 SISH signals is more straightforward than interpretation of HER2 IHC may play a role here. Contrary to ERα and PR that preferentially converted from positive in the primary tumors to negative in the metastases, HER2 receptor conversion occurred both ways. Although from a tumor progression model one would expect HER2 conversion to preferentially occur from negative to positive, conversion both ways has been described before [17,18,20-23]. One mechanism of conversion from positive to negative may well be explained by clonal selection of HER2 negative cells during the metastatic process, for example, elicited by trastuzumab therapy [39].

When considering the different metastatic sites, receptor conversion seemed to occur mostly in liver and brain metastases, but only for PR conversion this was significant. The reason for this observation is unclear, and these results need to be interpreted with caution.

One limitation to receptor conversion studies is the lack of internal control cells in samples from most of the metastatic sites. An exception to this is the liver that bears ERα and to a lesser extent PR expression in hepatocytes. Since most receptor conversion was seen in the liver, and the fact that most biopsies from breast cancer metastases are small and therefore probably quickly and well fixed, it is unlikely that these issues play an important role. A further limitation was the deliberate choice not to include the preferential metastatic site of breast cancer: the bone (marrow). This was to avoid false negative results due to decalcification artefacts. Such false negative results are not easy to trace since internal positive control cells in the bone marrow are lacking. Future studies selectively studying small bone biopsies that were not decalcified may shed further light on percentages of conversion on this metastatic site.

Nearly 11% of the patients converted from ER+ or PR+ to ER-/PR- and 3.4% from ER-/PR- to ER+ or PR+ (using the 10% threshold); in these cases steroid receptor conversion could be especially clinically relevant.

Together with the HER2 conversion rate of 5.2% by IHC, in about 19% of metastatic patients the choice of systemic therapy is suboptimal when solely based on IHC of the primary tumor. However, before concluding that metastases should be biopsied when possible, there are a few issues to consider. Clinicians would probably be inclined to treat patients with positively converted distant metastases (3.4 + 2.6 = 6%) with the matching (hormonal or trastuzumab) systemic treatment, but for patients with negatively converted distant metastases this is probably more complicated. First, technical problems in cases with receptor negative metastases cannot be fully excluded. Second, there may be heterogeneity between distant metastases from which only one may get biopsied [40]. Third, there are few clinical data on response to systemic treatment in negatively converted patients. Therefore, clinicians might be inclined to consider (hormonal or trastuzumab) systemic treatment even in negatively converted patients. Nevertheless, when a biopsy of a distant metastasis is available, hormone receptor and HER2 status should be reassessed in these biopsies and the tests results should be critically evaluated in conjunction with ER, PR and HER2 status of the primary tumor. In the future, non-invasive assessment of the receptor status by molecular imaging may form an alternate and more functional way of assessing receptor status of distant metastases [24-26], especially for metastases at inaccessible sites, also providing information on heterogeneity of receptor status between distant metastases.

## Conclusions

In conclusion, receptor conversion in distant non-bone breast cancer metastases indeed occurs, is relatively uncommon for ERα and HER2, more frequent for PR, and seems to be more frequent in liver and brain metastases. In a considerable number of patients such conversion could theoretically have consequences for the systemic therapeutic regimen. For this reason, receptor status should therefore be reassessed on available biopsies from distant metastases. Whether distant breast cancer metastases should be more routinely biopsied when possible, will likely be the subject of further discussion. In the future, non-invasive assessment of the receptor status by molecular imaging may be an attractive alternative, especially for metastases that are difficult to biopsy.

## Abbreviations

ASCO: American Society of Clinical Oncoloy; CAP: College of American Pathologists; EDTA: ethylenediaminetetraacetic acid; EQA: external quality assement; ERα: estrogen receptor alpha; HC: immunohistochemistry; HER2: human epidermal growth factor receptor 2; ISH: *in situ *hybridization; PBS: phosphate buffered saline; PET: positron emission tomography; PR: progesterone receptor; SISH: silver in situ hybridization; SPECT: single photon emission computed tomography; TMA: tissue microarrays.

## Competing interests

The authors declare that they have no competing interests.

## Authors' contributions

LDCH, MJvdV, PvdG, EvdW and PJvD made substantial contributions to the concept and design of the study. MJvdV, H-JvS, PW, JW, PJW, JB, CAS, IDN, JO, PvdV and PJvD were involved in the provision of study material. LDCH, MJvdV and PJvD were involved in the acquisition of data. LDCH, MJvdV and PJvD contributed to the analysis and interpretation of data. LDCH, MJvdV and PJvD wrote the manuscript. All authors critically reviewed the report and approved the final version of the report for submission.

The corresponding author (PJvD) had access to the primary data, took responsibility for accuracy and completeness of data reporting, and had final responsibility for the decision to submit for publication.
